# In Vitro Antibacterial and Antibiofilm Activity of* Lippia alba* Essential Oil, Citral, and Carvone against* Staphylococcus aureus*

**DOI:** 10.1155/2017/4962707

**Published:** 2017-08-03

**Authors:** Emanuela Mesquita Porfírio, Hider Machado Melo, Antônio Matheus Gomes Pereira, Theodora Thays Arruda Cavalcante, Geovany Amorim Gomes, Mário Geraldo de Carvalho, Renata Albuquerque Costa, Francisco Eduardo Aragão Catunda Júnior

**Affiliations:** ^1^INTA College, Cel. Antonio Rodrigues Magalhães, 359 Sobral, CE, Brazil; ^2^State University of Acaraú Valley, 62040-370 Sobral, CE, Brazil; ^3^Federal Rural University of Rio de Janeiro, 23890-000 Seropédica, RJ, Brazil; ^4^State University of the Tocantina Region of Maranhão, 65901-480 Imperatriz, MA, Brazil

## Abstract

In vitro antimicrobial and antibiofilm activities of the* Lippia alba* essential oil and its major components (citral and carvone) against* Staphylococcus aureus* were investigated. Essential oils (LA1EO, LA2EO, and LA3EO) were extracted from the aerial parts of three* L. alba* specimens by hydrodistillation and analyzed by gas chromatography coupled to a mass spectrometer. Minimum Inhibitory Concentrations (MIC) and Minimum Bacterial Concentration (MBC) were determined by the microdilution method. For the antibiofilm assays, the biomass formation in the biofilm was evaluated by the microtiter-plate technique with the crystal violet (CV) assay and the viability of the bacterial cells was analyzed. All oils and their major components presented antibacterial activity, and the lowest MIC and MBC values were 0.5 mg mL^−1^ when LA1EO and citral were used. Potential inhibition (100%) of* S. aureus* biofilm formation at the concentration of 0.5 mg mL^−1^ of all EOs was observed. However, the elimination of biofilm cells was confirmed at concentrations of 1 mg mL^−1^, 2 mg mL^−1^, 2 mg mL^−1^, and 0.5 mg mL^−1^ for LA1EO, LA2EO, LA3EO, and citral, respectively. The results obtained in the present research point to the promising antibacterial and antibiofilm potential of* L. alba* EOs against* S. aureus*, a species of recognized clinical interest.

## 1. Introduction

 Antimicrobial resistance in bacterial pathogens is a public health problem worldwide and is associated with high morbidity and mortality, jeopardizing the efficacy of antibiotics. In this sense,* S. aureus*, a Gram-positive bacterium recognized as a pathogenic agent, is associated with food poisoning [1, 2] and/or severe infection [3]. In this study, the use of resistant strains to methicillin [4] and vancomycin [5] has been highlighted.

This problem of bacterial resistance has been attributed to the excessive and incorrect use of drugs, as well as the lack of development of new drugs by the pharmaceutical industry, thus arising from the need to search for natural products that may come to combat these “superresistant bacteria” [6–9].

Natural products extracted from plants are identified as an alternative for the discovery of new active antimicrobial agents through empirical knowledge regarding the use of medicinal plants [10]. The use of antimicrobial phytotherapeutics is considered as a possible source of new antibiotic discoveries with a broad spectrum of actions different from traditional antibiotics [11].

Among the plants recognized for their pharmacological action are those belonging to the genus* Lippia*, which comprises about 200 species distributed in tropical, subtropical, and temperate regions of America, Africa, and Asia [12]. The species* L. alba* is a shrub of the Verbenaceae family, popularly known as lemon balm. Its essential oil is mentioned with a broad spectrum of activities against Gram-negative and Gram-positive bacteria, whose effectiveness varies according to its composition, which is influenced by biotic and abiotic factors [13].

Thus, the present study aims to evaluate the antibacterial and antibiofilm activity of essential oils of three* L. alba *chemotypes and of their major compounds (citral and carvone) against* S. aureus*.

## 2. Materials and Methods

### 2.1. Plant Material

Aerial parts of three chemotypes of* L. alba* were collected. The first was collected in July 2014, and the second was collected in June 2016, both at the Experimental Farm of the INTA College, located in the municipality of Cariré, northwest of Ceará state, Brazil, at 3°49′51 82′′S40°24′37 85′′W, at 76 meters from sea level, and the third one was collected in March 2016, in the Medicinal Plants Garden of Sumaré in the municipality of Sobral, northwest of Ceará state, Brazil, at 30 42′ 07 31′′S 400 21′ 53 66′′O, at 88 meters from sea level. The voucher specimens (numbers 17481, 20790, and 20441) were deposited in Prof. Francisco José de Abreu Matos Herbarium (HUVA) of the Center for Agricultural Sciences and Biological Sciences, State University of Acaraú Valley, Sobral, Ceará state, Brazil.

### 2.2. Extraction of Essential Oils

Aerial parts were air-dried, ground, and submitted to hydrodistillation (~1 kg, 3 h) using a Clevenger-type apparatus for LAEO obtention. Oils were dried with anhydrous Na_2_SO_4_ and transferred to glass flasks that were kept at −5°C until used. Yield of 0.578% w/w, 1.203% w/w, and 2.654% w/w was observed for LA1EO, LA2EO, and LA3EO, respectively.

### 2.3. Analysis of the Essential Oil

The chemical composition of the essential oil was analyzed according to Figueiredo et al. [14]. A gas chromatograph coupled to a mass spectrometer (GC/MS, Shimadzu QP-2010 Plus) equipped with a Factor Four/VF (5 ms) fused-silica capillary column (30 m × 0.25 mm × 0.25 *μ*m film thickness), with helium as carrier gas at 1 mL min^−1^, was used. The initial oven temperature was 35°C, which after being held constant for 2 min was increased at a rate of 4°C min^−1^ to 180°C, followed by 10°C min^−1^ to 250°C, with a final isotherm (250°C) for 20 min. The sample injection was 1 *μ* (1 : 50 split mode). The injector and detector temperatures were both 250°C. The mass spectra were obtained in a range of* m/z* 10–300, by the electron impact technique at 70 eV. The quantitative analysis of the oils' chemical composition was carried out in a gas chromatograph coupled to an HP5890 Series II flame ionization detector (FID), using the same operational conditions and the same type of column as in the GC/MS analysis, with the exception of the injector and detector temperatures that were 240 and 300°C, respectively. The percentage of each constituent was calculated by the integral area under the respective peaks in relation to the total area of all the sample constituents. The various chemical constituents of the essential oil were identified by visual comparison of their mass spectra with those in the literature [15] and spectra supplied by the equipment database (NIST 08), as well as by comparison of the retention indices with those in the literature [15]. A standard solution of *n*-alkanes (C8–C20) was injected under the same chromatographic conditions as the sample and used to obtain the retention indices as described by van Den Dool and Kratz [16].

### 2.4. Solutions and Drugs

Citral and carvone (Sigma-Aldrich, USA) were used to perform the microbiological tests.

### 2.5. Strain Origin

*Staphylococcus aureus* ATCC 6538 was used. This strain was stored in Skin Milk with 20% glycerol and was reactivated in Tryptone Soy Broth for bacteriological analyses.

### 2.6. Minimum Inhibitory Concentration (MIC)

MIC was determined by microdilution assay on 96-well polystyrene plates [17] with 20 replicates. To perform the test, the bacterial culture concentration was adjusted to 10^6^–10^8^ CFU mL^−1^ in Tryptic Soy Broth medium (Difco). The test substances (LA1EO, LA2EO, LA3EO, citral, and carvone) were tested at the concentrations of 4, 2, 1, 0.5, 0.25, 0.125, and 0.062 mg mL^−1^. 100 *μ*L of the adjusted culture was placed in contact with the test substances and the plates were incubated in an oven at 37°C for 24 h. MIC was considered the lowest concentration of the test substance where no microbial growth was visualized.

### 2.7. Minimum Bactericidal Concentration (MBC)

MBC was determined by the plating of 10 *μ*L (in triplicate) of the well contents of the plates used in the MIC (after the incubation period) in Tryptone Soy Agar (Difco). CBM was considered the lowest concentration of the test substance in which no microbial growth was observed after the incubation period (37°C for 24 h).

### 2.8. Antibiofilm Activity

The biofilm biomass formation was evaluated using the microtiter-plate technique with the crystal violet (CV) assay [18], and the viability of the bacterial cells in the biofilm was analyzed [19]. The biofilms associated with the surface of the wells were fixed with methyl alcohol and stained with 0.1% crystal violet for fifteen minutes. After this period, each plate was washed (TriContinent's MultiWash III) and subjected to absorbance reading in a microplate reader (SpectraMax) at 595 nm. In order to verify the viability of the bacterial cells in the biofilm (10^−1^ to 10^−6^), the plates were submitted to an ultrasonic bath (GNATUS Digital Ultrasonic Cleaner) for 5 minutes, which were plated (10 *μ*L in triplicate) on Tryptic Agar Soy (Difco), and were incubated at 37°C for 24 h. After the incubation period, colony forming units (CFU mL^−1^) were determined.

### 2.9. Controls

For the CIM, CBM, and antibiofilm activity, we used as turbidity control the culture medium (TSB) containing the test substance (LA1EO, LA2EO, LA3EO, citral, and carvone) at concentrations of 4 mg mL^−1^, 2 mg mL^−1^, 1 mg mL^−1^, 0.5 mg mL^−1^, 0.25 mg mL^−1^, 0.125 mg mL^−1^, and 0.0625 mg mL^−1^. For contamination control, only the culture medium (TSB) in three wells on the plates was used. The negative control was made from the inoculation of 100 *μ*L of strain (1.25 × 10^7^ CFU mL^−1^) in wells containing culture medium (TSB). As a positive control, vancomycin was used in the same concentrations of the test substances: 4 mg mL^−1^, 2 mg mL^−1^, 1 mg mL^−1^, 0.5 mg mL^−1^, 0.25 mg mL^−1^, 0.125 mg mL^−1^, and 0.0625 mg mL^−1^.

### 2.10. Statistical Analysis

ANOVA (analysis of variance) was used followed by the Student-Newman-Keuls test with 95% confidence interval (*p* < 0.05).

## 3. Results and Discussion

**Figure 1:**
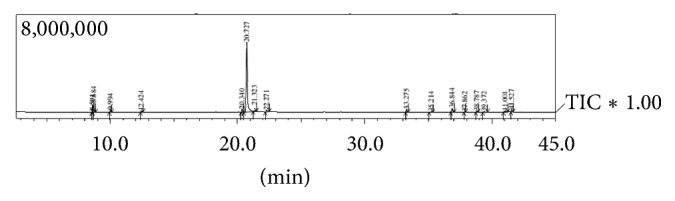
GC-MS chromatogram of the essential oil of* Lippia alba*: LA3 chemotype.

The results of the LA1EO, LA2EO, and LA3EO analyses are described in Table 1 and Figure 1. The chemical constituents that obtained concentrations above 10% were considered as the major constituents. For LA1EO, 25 components were identified, with predominance of geranial (35.82%), neral (26.44%), and* p*-cymene (9.84%). LA2EO presented 23 identified components, with geranial (24.87%), neral (18.42%), and geranic acid (17.24%) being the most prevalent. In LA3EO, 13 components were identified, with carvone (71.54%) being the major one, followed by limonene (5.25%) and geranial (4.66%).

Due to the absence of established standardization for the differentiation of chemotypes in* Lippia alba*, many authors relied on the similarity between the major compounds present in the specimens of the species [20]. In the present study, the presence of these major compounds (citral and carvone) makes it possible to classify LAEO as three* L. alba* chemotypes.

According to Mamun-Or-Rashid et al. [12], at least 12* Lippia *chemotypes have been described according to the major chemical components, namely, citral, linalool, carvone, limonene, *γ*-terpinene, citral-myrcene, citral-limonene, citral-caryophyllene, citral-germacrene D, carvone-limonene, 1,8-cineole-camphor, 1,8-cineol-limonene, and limonene-piperitone, and there are predominantly monoterpene-like compounds such as citral, myrcene, and limonene.

The phytochemical analysis obtained for EOLA (Table 1) was similar to that reported by Machado et al. [13] that identified citral (31.6%) and neral (25.5%) as the main components of the LAOE. This indicates that the plant belongs to the “citral” chemotype composed of neral + geranial [21]. Similar values were also demonstrated in studies by Gonçalves et al. [22] (neral: 28.29%; geranial: 38.88%), and chemotype III (neral: 22.49%; geranial: 34.64%) and chemotype II (neral: 28.29%; geranial: 38.88%) were analyzed (carvone: 62.30%; limonene: 16.31%).

The antimicrobial activity of the oils and their major components against* S. aureus* was confirmed by the values of MIC and MBC. The lowest MIC value (0.5 mg mL^−1^) was observed when LA1EO, LA3EO, and citral were used. For MBC, the lowest value was 0.5 mg mL^−1^ when LA1EO and citral were used (Table 2). Essential oils are hydrophobic compounds and easily diffuse through the cell wall of microorganisms, causing damage to the membrane, especially with respect to fluidity and permeability [23].

In this study, MIC and MBC values were the same for LA1EO and citral, both presenting bacteriostatic and bactericidal action at the concentration of 0.5 mg mL^−1^. LA2EO showed MIC and MBC at 1 mg mL^−1^ concentration (2x higher concentration than LA1EO and citral). On the other hand, LA3EO showed an inhibitory action at the same concentration as LA1EO and citral. Carvone was able to reach inhibitory action only at the concentration of 2 mg mL^−1^, that is, at a concentration 4 times higher than that of the LA1EO, LA2EO, and citral oils. It can also be verified that the LA3EO presented bactericidal action only at the concentration of 2 mg mL^−1^ (concentration 4x greater than that of LA1EO and citral and 2x greater than that of LA2EO). Our results show that the planktonic* S. aureus* ATCC 6538 has different susceptibility to EOs of* L. alba* and its major components, suggesting that the bacteriostatic and bactericidal actions are due to the synergistic action of the components of the oils and not only the action of the majority component.

The values obtained for CIM and CBM (Table 2) were lower than those reported by Sutili et al. [24] that demonstrated bacteriostatic and bactericidal action against* S. aureus* from the concentration of 2.8 mg mL^−1^ and 5.9 mg mL^−1^ LAEO, respectively. On the other hand, our results resemble those of Machado et al. [13] who presented MIC and MBC values at concentrations of 0.29 mg mL^−1^ and 0.33 mg mL^−1^, respectively, validating their antimicrobial potential.

**Figure 2:**
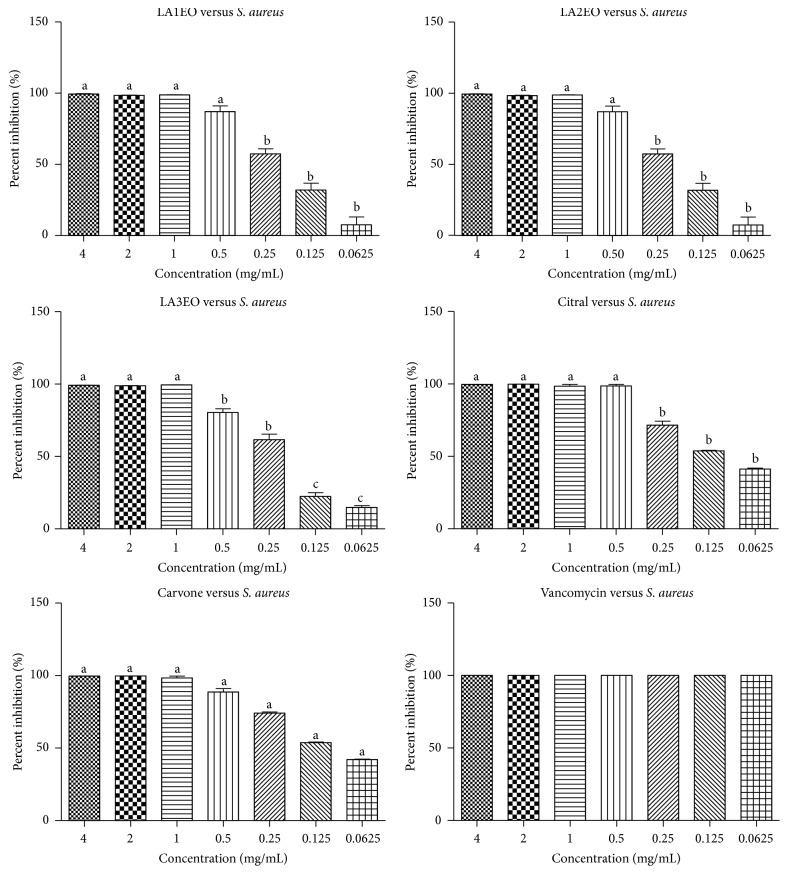
Inhibition (%) of* Staphylococcus aureus* ATCC 6538 biofilm formation by essential oils of* Lippia alba* (LA1EO, LA2EO, and LA3EO), citral, carvone, and vancomycin in concentrations of 4, 2, 1, 0.5, 0.25, 0.125, and 0.062 mg mL^−1^. ANOVA followed by Student-Newman-Keuls test; *p* < 0.001 (a) versus all variables; *p* < 0.001 (b) versus (c).

All EOLA presented inhibition potential (100%) of* S. aureus* (Figure 2) at the concentration of 0.5 mg mL^−1^ of LA1EO, LA2EO, citral, and carvone. In addition, potential inhibition was observed in the three concentrations below 0.5 mg mL^−1^ of LAEOs, citral, and carvone. The potential of biofilm formation of concentrations 0.25, 0.125, and 0.0625 mg mL^−1^ was lower than that observed in the positive control, vancomycin, which is the drug of choice for the treatment of infections caused by MRSA.

The antibiofilm activity of LAEOs should be highlighted, since this* S. aureus* is a successful human pathogen due to its metabolic versatility and its ability to adapt to host defensive stress [25]. This pathogen can cause mild infections and life-threatening diseases, including skin and soft tissue infections, bacteremia, pneumonia, endocarditis, septicemia, and toxic shock syndrome [26].

**Figure 3:**
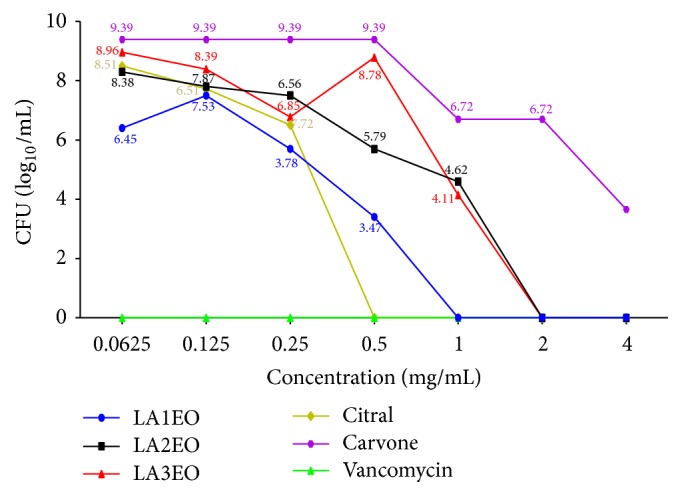
Colony forming units (CFU log_10 _mL^−1^) count of biofilm cells of* Staphylococcus aureus *ATCC 6538 after 24-hour treatment with essential oils of* Lippia alba* (LA1EO, LA2EO, and LA3EO), citral, carvone, and vancomycin at concentrations of 4, 2, 1, 0.5, 0.25, 0.125, and 0.062 mg/mL^−1^.

Colony forming units count revealed that the elimination of biofilm cells was confirmed at concentrations of 1 mg/mL, 2 mg/mL, 2 mg/mL, and 0.5 mg/mL for LA1EO, LA2EO, LA3EO, and citral, respectively (Figure 3). No elimination of biofilm cells was observed when carvone was used (Figure 3).

In the present study, the antibiofilm action of OELA1 and OELA2 seems to be related to the presence of citral, since this isolated component presented the best results. Citral, a monoterpene (3,7-dimethyl-2,6-octadiene), is a combination of two neral and geranial isomers [27] and has a strong antimicrobial activity [28, 29].

The antibiofilm effect of LA3EO resulted from the synergistic action of its constituents and not only from the action of carvone, its major component, which was not able to completely eliminate the cells of the biofilm in formation. Carvone is a monoterpene found as the main active component of several essential oils, such as mint (*Mentha spicata* L.), dill (*Anethum graveolens* L.), caraway (*Carum carvi* L.), and lemon grass (*Lippia alba*) [30]. Znini et al. [31] reported that carvone is one of the most effective antimicrobial agents of several plants, with a mechanism of antibacterial activity involving the destabilization of the structure of phospholipid and the interaction with membrane proteins, and acts as a proton exchanger reducing the pH gradient across the membrane [32].

Thus, our results point to the in vitro effect of the essential oils of three specimens of* L. alba* against planktonic and biofilm of* S. aureus*, a recognized pathogen of clinical interest.

## Figures and Tables

**Table 1:** Chemical composition, calculated retention index (RI_C_), percentages of identified components (%), and similarity index (SI) in comparison with MS library, of essential oils from *Lippia alba* chemotypes: LA1, LA2, and LA3.

Component	RI_C_	LA1	SI	LA2	SI	LA3	SI

Sabinene	975	0.54	93	—^a^	—	—	—
6-Methyl-5-hepten-2-one	985	3.89	96	2.34	94	—	—
Myrcene	990	4.08	96	—	—	—	—
*p*-Cymene	1024	**9.84**	95	4.12	96	0.96	91
Limonene	1029	—	—	1.64	95	5.25	94
*γ*-Terpinene	1059	0.41	95	—	—	1.70	93
*E*-4-Thujanol	1070	0.58	94	1.23	90	—	—
*Z*-Linalool oxide	1072	—	—	0.13	91	—	—
*E*-Linalool oxide	1086	—	—	0.27	92	—	—
Linalool	1096	1.16	96	1.02	94	1.38	91
*Z*-Limonene oxide	1136	—	—	0.75	88	—	—
*E*-Limonene oxide	1142	—	—	1.12	89	—	—
*E*-Isocitral	1180	0.39	96	—	—	—	—
Citronellol	1225	—	—	2.30	85	—	—
Nerol	1229	1.64	95	—	—	—	—
Neral	1238	**26.44**	97	**18.42**	96	2.88	95
Carvone	1243	—	—	4.25	94	**71.54**	97
Piperitone	1252	—	—	—	—	0.73	90
Geraniol	1252	1.86	96	—	—	—	—
Geranial	1267	**35.82**	97	**24.87 **	97	4.66	95
Hydroxycitronellal	1288	—	—	2.73	76	—	—
Neric acid	1340	0.42	92	5.19	84	—	—
Geranic acid	1359	0.36	96	**17.24**	95	—	—
Germacrene D	1485	—	—	—	—	2.51	92
Cubebol	1515	—	—	—	—	0.92	73
Elemol	1549	5.41	95	5.04	94	3.90	93
Germacrene D-4-ol	1575	—	—	—	—	0.88	72
Caryophyllene oxide	1583	0.95	92	—	—	—	—
Guaiol	1600	1.49	91	—	—	0.92	87
(Citral = neral + geranial)		(62.26)	—	(43.29)	—	(7.54)	—

Total		95.28	—	92.64	—	98.23	—

^a^Not detected.

**Table 2:** Minimum Inhibitory Concentration (MIC) and Minimum Bactericidal Concentration (MBC) of *Lippia alba *essentials oils (LA1EO, LA2EO, and LA3EO), citral, and carvone against *Staphylococcus aureus.*

Substance	MIC (mg mL^−1^)	MBC (mg mL^−1^)
LA1EO	0.5	0.5
LA2EO	1.0	1.0
LA3EO	0.5	2
Citral	0.5	0.5
Carvone	2.0	—
